# Effects of Different Methionine Levels in Low Protein Diets on Production Performance, Reproductive System, Metabolism, and Gut Microbiota in Laying Hens

**DOI:** 10.3389/fnut.2021.739676

**Published:** 2021-10-06

**Authors:** Miaolin Ma, Shunju Geng, Meiling Liu, Lihong Zhao, Jianyun Zhang, Shimeng Huang, Qiugang Ma

**Affiliations:** State Key Laboratory of Animal Nutrition, College of Animal Science and Technology, China Agricultural University, Beijing, China

**Keywords:** laying hens, methionine deficiency, low protein diets, gut microbiota, production performance, nutrient metabolism

## Abstract

This study investigated the effects of different levels of methionine (Met) in a low protein diet on the production performance, reproductive system, metabolism, and gut microbial composition of laying hens to reveal the underlying molecular mechanism of Met in a low protein diet on the host metabolism and gut microbial composition and function of hens. A total of 360 healthy 38-week-old Peking Pink laying hens with similar body conditions and egg production (EP) were randomly divided into four groups with nine replicates per treatment and 10 hens per replicate. The hens in each treatment group were fed low protein diets containing different levels of Met (0.25, 0.31, 0.38, and 0.47%, respectively) for 12 weeks. Feed and water were provided *ad libitum* throughout the trial period. The results showed that, compared with the 0.25% Met group, the final body weight (FBW), average daily gain (ADG), EP, egg weight (EW), and average daily feed intake (ADFI) in the other groups were significantly increased and feed egg ratio (FER) was decreased. Meanwhile, the EW and yield of abdominal fat (AFY) in the 0.47% Met group were higher than those in other groups. The triglyceride (TG), estradiol (E2), total protein (TP), albumin (ALB), and immunoglobulin A (IgA) in the 0.38 and 0.47% Met groups were higher than those in other groups. In addition, 16S rRNA gene sequencing revealed that there was no difference in the Sobs index, ACE index, and Shannon index among all groups. However, it is worth noting that feeding low protein diets with Met changed the gut microbial composition (e.g., the supplementation of Met increased the level of *Lactobacillus* and decreased the proportion of *Faecalibacterium*). Also, our results showed that the changes in gut microbial composition induced by the diets with different levels of Met were closely related to the changes of key parameters: ADFI, EW, FBW, TG, EM, EP, ADG, FER, and uric acid (UA). Our results highlight the role of adding an appropriate amount of Met to the low protein diet in laying hens, which could improve the gut microbial composition, production performance, reproductive system, and nutrient metabolism of laying hens. In conclusion, this study suggested that when the Met level was 0.38%, the production performance of the laying hens was pretty good.

## Introduction

The poultry industry shows a trend of large-scale, intelligent, and standardized development, and the demand for protein feed resources continues to increase (1, 2). However, there are problems, such as limited protein resources, low nutrient utilization rates, and environmental pollution, caused by fecal nitrogen emission, thereby making the research on the low protein diet is becoming more and more important. In order to ensure the performance of animals, a large amount of protein is added to the diet of laying hens. However, excessive protein in the diet, which will increase the content of nitrogen and phosphorus in feces and urine, is often not put to good use (3, 4). Excessive ammonia nitrogen emission in feces will not only cause environmental pollution, but also harm the health of laying hens (5–8). Some studies have shown that reducing protein levels in the diet can effectively reduce nitrogen emissions from the feces and urine of livestock and poultry (9–11).

That refers to the low protein diet that containing less crude protein than the nutrient requirements of poultry (NRC) recommends for feeding in the diet. Following the ideal amino acid pattern is the key to feed formulation design. In essence, the need for protein in the diet is the need for amino acids, which are essential nutrients for animal growth and development that are involved in important biochemical reactions in animals (12, 13). There have also been studies on livestock and poultry that have shown that adding certain synthetic amino acids to a low protein diet can improve protein digestibility, effectively reduce nitrogen emissions (11, 14), and save feed costs (15, 16). Many studies have shown that adding a certain amount of glycine, valine, isoleucine, and methionine (Met) to a low protein diet has no negative effect on livestock and poultry and can even improve their performance in some way (17–19). In addition, it can also improve the structure and composition of the gut microbiota of livestock and poultry, increase the abundance of beneficial microbes such as *Bifidobacterium* and *Lactobacillus*, maintain the integrity of the intestinal epithelial barrier function, improve the normal functions of the intestinal epithelium, and enhance intestinal mucosal immunity in animals (20–23).

In order to avoid the negative effects of a low protein diet on livestock and poultry, we can consider adding essential amino acids to their diet to reduce harmful effects (24). Methionine, as the main limiting amino acid in the corn-soybean meal diet of laying hens, not only participates in protein synthesis, but also plays an irreplaceable role in some molecular precursors and intermediates to control oxidative stress in the body and affect cell metabolism and function (25). It was also found that dietary Met can enhance the immune response levels of poultry (26, 27). Furthermore, Met can be added into the low protein diet of broilers to meet the requirements of total sulfur-containing amino acids, but it should be added appropriately, otherwise, it will affect the growth performance of poultry (28). Zhang found that supplemented Met in the diet could change the intestinal tissue morphology and increase the body weight of Peking ducks (29). In addition, Met can provide methyl (30, 31), and use it for the methylation of toxic substances to detoxify. It can also combine with mycotoxin to weaken its toxicity. But there is still a lack of research on the effect of Met supplementation in low protein diets on laying hens, especially on the effect of gut microbiota.

Due to the current situation, low protein diets will be a trend in the future. However, the application of a low crude protein diet in laying hens is relatively rare at present, and the study on its functional effect on laying hens can better prepare balanced low protein diets, which is conducive to exerting the genetic potential of laying hens. To develop a practical understanding of Met use in low crude protein diets, a study was conducted to evaluate production performance, egg quality, reproductive system, host metabolism, and the gut microbial composition responses to different levels of Met in laying hens fed low protein diets.

## Materials and Methods

### Animal Experimental Ethics

All experiments were approved by the China Agricultural University Animal Care and Use Committee (AW32301202-2-1, Beijing, China).

### Animals and Experimental Design

A total of 360 commercial hens of the Peking Pink strain (Yukou Poultry Co., Ltd., Beijing, China) at the age of 38 weeks with similar egg production and healthy bodies were randomly divided into four treatment groups with nine replicates per group and 10 birds per replicate. The hens were placed in five cages (two birds in each cage) and each cage (H45 cm × W45 cm × D45 cm) was equipped with a nipple drinker and an exterior feed through to ensure feed and water were provided *ad libitum* during the entire experimental period. At the same time, to ensure that the chicken coop is closed and ventilated, the average relative humidity was routinely maintained at ~55%, and it was ensured that the hens get 16 h of light every day. In order to meet the nutritional requirements of the laying hens (NYT33-2004), a basal corn-soybean meal diet was formulated. A pre-experiment was conducted for one week before the start of the formal experiment to ensure that the animals were acclimated to the new experimental environment and diet, meanwhile, it could empty the original intestinal contents, and estimate the approximate feed intake of the experimental animals. The four experimental groups were fed with low protein diets containing 0.25, 0.31, 0.38, and 0.47% Met (0.25% Met group, 0.31% Met group, 0.38% Met group, and 0.47% Met group), respectively. The ingredients and nutrient composition of the diets are shown in Supplementary Table 1.

### Laying Performance and Egg Quality

The difference between the full bucket weights and the remaining feed was calculated as the weekly feed intake, and the body weights of the laying hens were recorded every week at the same time to calculate the average daily feed intake (ADFI) and feed egg ratio (FER). The number of eggs and egg weight were accurately recorded every day to calculate the average daily egg mass (EM), egg weight (EW), and egg production (EP) rate. The hens were weighed in replicates at the beginning and end of the experiment to calculate average daily gain (ADG). After the beginning of the experiment, 30 eggs were randomly selected from each treatment group every 4 weeks to measure egg quality parameters as shown in **Table 2**.

The eggshell strength was measured by the egg force reader (ESTG-01COrka Teachnology Ltd); The eggshell thickness was measured using the eggshell thickness tester (ESTG-01, Orka Teachnology Ltd); Haugh unit, yolks color, and egg weight were measured by multifunctional egg quality tester (EA-01, Orka Technology Ltd). The eggshell color was measured by QCR color reflectometer (QCR SPA, TSS England). Weigh the eggshell, then separate the yolk with a separator, and then weigh the ratio of yolk and the ratio of albumen.

### Blood Sampling and Biochemical Analysis

At the end of the experiment, blood samples were collected from the wing veins of the laying hens on the same day of sampling and centrifuged at 3,000 rpm for 15 min at room temperature to separate the serum. After that, the serum samples were collected by a pipette into 1.5-ml tubes and stored at −20°C. Triglyceride (TG), uric acid (UA), urea, total protein (TP), albumin (ALB), and immunoglobulin M (IgM) in serum were determined using an automatic biochemical analyzer (7600, Hitachi, Japan) according to the manufacturer's instructions. Superoxide dismutase (SOD) and glutathione (GSH) in the serum were determined using a commercial kit (Nanjing Jiancheng Bioengineering Institute, Nanjing, China) according to the kit instructions.

### Collecting Samples

Seventy hens were euthanized and weighed by a sodium pentobarbital injection (0.4 ml/kg·BW; Sile Biological Technology Co., Ltd., Guangzhou, China). Abdominal adipose tissue was weighed to calculate the yield of abdominal fat (AFY). The liver, kidneys, fallopian tubes, and ovaries were removed, weighed, and the number of follicles was recorded to calculate the liver index, fallopian tube index, and ovary index.

### DNA Extraction, Amplification, and Sequencing

The cecal contents of laying hens were collected, immediately frozen in liquid nitrogen, and stored at −80°C. Cecal microbial DNA was isolated with an Omega Bio-tek stool DNA kit (Omega, Norcross, GA, USA) and quantified by a NanoDrop 2000 spectrophotometer (Thermo Scientific, Waltham, MA, USA). Then, the V3–V4 region of the 16S rRNA gene was amplified with 338F and 806R primers with the sequence of 5′-ACTCCTACGGGAGCAGCA-3′ and 5′-GGACTACHVGGGTWTCTAAT-3′. Afterward, DNA samples were quantified, followed by the amplification of V3V4 hypervariable region of the 16S rDNA. Final amplicon pool was evaluated by the AxyPrep DNA gel extraction kit (Axygen Biosciences, Union City, CA, USA). Paired-end reads were generated with an Illumina MiSeq PE250 (Shanghai MajorBio Biopharma Technology Co., Ltd., Shanghai, China), and the reads were filtered out with default parameters.

### Statistical Analysis

All results were subjected to a one-way ANOVA procedure and differences were examined using Duncan's multiple range test to evaluate the differences within treatments using SPSS version 18.0 (SPSS Institute Inc., Chicago, USA). The trends of the linear and quadratic analyses were conducted using SAS software version 8.0 (version 9.2, SAS Institute Inc., Cary, NC, USA). Differences were considered significant at *p* < 0.05. Data were expressed as the mean ± SE.

The raw paired-end reads were assembled into longer sequences, and quality was filtered by PANDAseq (version 2.9) to remove the low-quality reads. The high-quality sequences were clustered into operational taxonomic units (OTUs) with a 97% similarity using UPARSE (version 7.0) in QIIME (version 1.17) (32, 33), and the chimeric sequences were removed using UCHIME (34). Taxonomy was assigned to OTUs using the RDP classifier. The subsequent clean reads were clustered as OTUs using UPARSE (version 7.0) and annotated with the SILVA 16S rRNA gene database using the MOTHUR program (version v.1.30.1) (35). Alpha-diversity (the Chao index, Ace index, and Sob index) was calculated based on the profiles of OTU by the MOTHUR program (36). Bar plots and heat maps were generated with the “vegan” package in R (version 3.3.1). A principal coordinate analysis (PCoA) was performed based on the Bray–Curtis distance using QIIME (version 1.17). An analysis of similarities (ANOSIM) was performed to compare the similarity of bacterial communities among groups using the “vegan” package of R (version 3.3.1). A linear discriminant analysis (LDA) effect size (LEfSe) was performed to identify the bacterial taxa that are differentially enriched in different bacterial communities. In a redundancy analysis (RDA), the variance between the samples (genus-level relative bacterial abundance) is explained by the phenotype of laying hens, which were fitted to corresponding matrices in the resulting illustration (37–39). Phylogenetic Investigation of Communities by Reconstruction of Unobserved States (PICRUSt) was also used to obtain a deeper insight into different pathways based on the Kyoto Encyclopedia of Genes and Genomes (KEGG) orthology between the four groups (40). Finally, the correlations between key parameters and bacterial communities were assessed by Spearman's correlation analysis using the “pheatmap” package in R (version 3.3.1). Data were expressed as mean values.

## Results

### Effects of Different Levels of Met Supplementation on the Laying Performance of Laying Hens With Low Protein Diets

During the experiment period (Table 1), dietary Met levels had significant positive effects on the performance of laying hens. There were no significant differences in FBW, ADG, EP, EW, EM, and ADFI between the 0.31, 0.38, and 0.47% Met groups, but they were significantly higher than those in the 0.25% Met group (*p* < 0.001). On the contrary, the FER in the 0.25% Met group was significantly higher than the other three groups (*p* < 0.001), while there was no significant difference among the other three groups. The AFY in the 0.47 and 0.38% Met groups was significantly higher than that in the 0.25% Met group (*p* < 0.01). The EW in the 0.38% Met group was significantly lower than that in the 0.47% Met group (*p* < 0.001), the liver index was lower than that in the 0.25% Met group (*p* < 0.05), and IBW was not significantly affected by different dietary Met levels (Table 1).

**Table 1:** Effects of different dietary Met supplementation in low protein diets on the growth and laying performance of laying hens that are 38–50 weeks.

**Indexes**	**0.25%**	**0.31%**	**0.38%**	**0.47%**	**SE^d^**	***P-*value**	**Linear *P-*value**	**Quadratic *P*-value**
	**Met group**	**Met group**	**Met group**	**Met group**				
IBW (g)	1,507.3	1,507.6	1,502.9	1,512	15.77	0.982	0.896	0.78
FBW (g)	1,509.9^b^	1,647.2^a^	1,629.2^a^	1,652.9^a^	17.94	<0.001	<0.001	0.003
ADG (g/d)	0.03^b^	1.70^a^	1.54^a^	1.72^a^	0.19	<0.001	<0.001	<0.001
EP (%)	68.1^b^	83.9^a^	87.4^a^	86.9^a^	1.47	<0.001	<0.001	<0.001
EW (g)	56.4^c^	60.6^a^^,^^b^	60.1^b^	61.7^a^	0.39	<0.001	<0.001	0.002
EM (g/d)	38.4^b^	50.8^a^	52.5^a^	53.5^a^	0.87	<0.001	<0.001	<0.001
ADFI (g/d)	97.4^b^	110.2^a^	109.6^a^	110.7^a^	1.3	<0.001	<0.001	<0.001
FER	2.54^a^	2.17^b^	2.09^b^	2.07^b^	0.03	<0.001	<0.001	<0.001
AFY (%)	1.32^b^	2.52^a^^,^^b^	3.29^a^	3.08^a^	0.36	0.002	0.001	0.055
Liver index (%)	2.64^a^	2.35^a^^,^^b^	2.23^b^	2.29^a^^,^^b^	0.1	0.037	0.014	0.104

a,b,c *Means within a column with no common superscripts differ (p < 0.05)*.

d *Pooled SEM*.

When the diet was supplemented with 0.38% Met, the maximum EP (88.83%) was obtained. When the Met level was increased to 0.41%, the maximum EM (54.32 g/d) and minimum FER (2.04) were obtained, and the Met intake was 0.45, 0.46, and 0.46 g/d, respectively (Supplementary Table 2). When the dietary sulfur amino acid content was 0.62%, the maximum EP (88.28%) was obtained. When the diet contained 0.63% sulfur amino acids, the maximum EM (53.98 g/d) and the minimum FER (2.05) were obtained. At this time, the intake of sulfur amino acids was 0.69, 0.7, and 0.7 g/day, respectively (Supplementary Table 2).

### Effects of Different Levels of Met Supplementation on the Egg Quality of Laying Hens With Low Protein Diets

As shown in Table 2, the EW in the 0.25% Met group at 42 and 50 weeks of age was significantly lower than that of the other three groups (*p* < 0.001). The EW at 46 weeks of age in the 0.25% Met group was lower than that in the 0.31 and 0.47% Met groups (*p* < 0.05). The eggshell proportion at 46 weeks of age in the 0.25% Met group was higher than that in the 0.31% Met group (*p* < 0.05). The albumen ratio at 42 weeks of age in the 0.25% Met group was lower than that in the 0.31% Met group (*p* < 0.05), while the albumen ratio at 50 weeks of age was lower than that in the 0.47% Met group (*p* < 0.05). On the contrary, the yolk ratio at 42 weeks of age in the 0.25% Met group was higher than that in the 0.31% Met group (*p* < 0.05). The eggshell thickness in the 0.38% Met group at 46 weeks of age was significantly higher than that in the other three groups (*p* < 0.01), and there was no significant difference among the other three groups. The yolk color value in the 0.31% Met group at 50 weeks of age was the lowest and lower than that of the 0.25% Met group (*p* < 0.05). However, other indicators such as eggshell color, eggshell strength, and Haugh unit were not significantly affected by the different levels of dietary Met (Table 2).

**Table 2:** The effects of different dietary met supplementation in low protein diets on the egg quality of 42-, 46-, and 50-week-old laying hens.

**Indexes**	**Age**	**0.25%**	**0.31%**	**0.38%**	**0.47%**	**SE^c^**	***P-*** **value**		
		**Met group**	**Met group**	**Met group**	**Met group**		**Total**	**Linear**	**Quadratic**
Egg weight (g)	42	55.90^b^	59.94^a^	60.24^a^	61.90^a^	0.73	<0.001	<0.001	0.102
	46	58.47^b^	61.67^a^	61.20^a^^,^^b^	61.70^a^	0.84	0.019	0.014	0.107
	50	56.48^b^	60.23^a^	60.60^a^	61.61^a^	0.76	<0.001	<0.001	0.073
Shell (%)	42	10.94	10.81	10.8	10.63	0.15	0.523	0.154	0.922
	46	10.95^a^	10.10^b^	10.93^a^^,^^b^	10.70^a^^,^^b^	0.23	0.033	0.930	0.177
	50	10.8	10.8	10.72	10.37	0.19	0.345	0.118	0.366
Albumen (%)	42	62.36^b^	63.68^a^	63.53^a^^,^^b^	63.36^a^^,^^b^	0.32	0.025	0.056	0.026
	46	63.56	65.55	63.17	62.83	1.06	0.270	0.340	0.280
	50	61.37^b^	62.95^a^^,^^b^	62.32^a^^,^^b^	63.62^a^	0.54	0.026	0.012	0.79
Yolk (%)	42	26.69^a^	25.50^b^	25.68^a^^,^^b^	26.01^a^^,^^b^	0.28	0.018	0.138	0.008
	46	25.53	24.20	25.82	26.47	1	0.417	0.315	0.323
	50	27.82	26.27	26.96	26.01	0.52	0.071	0.045	0.560
Shell color	42	0.58	0.57	0.56	0.55	0.01	0.337	0.070	0.913
	46	55.89	56.53	54.82	56.98	0.95	0.417	0.711	0.424
	50	0.46	0.46	0.46	0.44	0.01	0.101	0.135	0.045
Shell thickness (mm)	42	0.58	0.57	0.56	0.55	0.01	0.337	0.070	0.913
	46	0.46^b^	0.46^b^	0.48^a^	0.46^b^	0.01	0.001	0.189	0.229
	50	0.46	0.46	0.46	0.44	0.01	0.101	0.135	0.045
Shell strength (N)	42	41.02	42.72	40.49	39.75	1.27	0.384	0.286	0.332
	46	40.98	40.9	41.25	40.61	1.32	0.989	0.896	0.835
	50	38.25	40.5	38.13	36.57	1.48	0.326	0.267	0.204
Haugh unit	42	84.49	83.23	82.96	83.47	1.03	0.740	0.471	0.388
	46	85.85	84.00	80.41	83.38	1.80	0.216	0.168	0.182
	50	82.23	85.58	81.61	82.53	1.89	0.466	0.717	0.523
Yolk color	42	4.86	4.77	4.83	4.60	0.10	0.240	0.100	0.500
	46	4.78	4.71	4.80	4.83	0.11	0.897	0.630	0.680
	50	4.73^a^	4.27^b^	4.43^a^^,^^b^	4.70^a^^,^^b^	0.13	0.029	0.906	0.004

a,b *Means within a column with no common superscripts differ (p < 0.05)*.

c *Pooled SEM*.

### Effects of Different Levels of Met Supplementation on the Host Metabolism of Laying Hens With Low Protein Diets

The different levels of dietary Met had significant effects on a number of biochemical indices (e.g., TG, ALB, UA, E2, and IgA) in the serum of laying hens. In particular, the TG and E2 in the 0.47% Met group were significantly higher than those in the 0.31% Met group (*p* < 0.01). Furthermore, IgA was higher than that in the 0.31% Met group (*p* < 0.05), while the TG, ALB, and E2 in the 0.38% Met group were significantly higher than those in the 0.31% Met group (*p* < 0.01). The TG in the 0.25% Met group was significantly lower than that in the 0.38 and 0.47% Met groups (*p* < 0.001), and UA was higher than that in the 0.31 and 0.47% Met groups (*p* < 0.05). However, other biochemical indices were not significantly affected by different dietary Met levels (e.g., UREA, TP, P, IgG, and IgM) (Table 3).

**Table 3:** The effects of different dietary Met supplementation in low protein diets on the serum parameters of laying hens that are 38–50 weeks.

**Indexes**	**0.25%**	**0.31%**	**0.38%**	**0.47%**	**SE^c^**	***P*-value**	**Linear**	**Quadratic**
	**Met group**	**Met group**	**Met group**	**Met group**			***P-*value**	***P*-value**
TG (mmol/L)	7.64^b^	8.51^b^	10.56^a^	11.54^a^	0.39	<0.001	<0.001	0.89
UA (μmol/L)	82.33^a^	65.00^b^	67.22^a^^,^^b^	64.56^b^	4.48	0.03	0.02	0.11
Urea (mmol/L)	0.31	0.32	0.33	0.37	0.02	0.31	0.09	0.45
TP (g/L)	37.47	35.23	39.52	39.28	1.6	0.22	0.18	0.54
ALB (g/L)	12.81^a^^,^^b^	11.41^b^	13.62^a^	13.00^a^^,^^b^	0.42	0.006	0.15	0.36
P (ng/mL)	0.84	0.84	1.05	1.13	0.19	0.61	0.21	0.85
E3 (pg/mL)	101.03^a^^,^^b^	83.91^b^	165.41^a^	172.78^a^	18.74	0.003	0.001	0.52
IgG (g/L)	5.98	6.19	6.08	6.53	0.27	0.52	0.21	0.67
IgA (g/L)	1.06^a^^,^^b^	0.99^b^	1.04^a^^,^^b^	1.12^a^	0.03	0.04	0.09	0.02
IgM (g/L)	0.8	0.83	0.79	0.89	0.03	0.15	0.11	0.32

a,b *Means within a column with no common superscripts differ (p < 0.05)*.

c *Pooled SEM*.

### Effects of Different Levels of Met Supplementation on the Reproductive System of Laying Hens With Low Protein Diets

As shown in Table 4, the different levels of dietary Met have extremely significant effects on tubal weight. The tubal weight of laying hens in the 0.47% Met group was significantly higher than that in the 0.25% Met group (*p* < 0.01) but it had no significant effects on oviduct length, ovary weight, and ovary weight after the removal of dominant follicles, etc., (Table 4).

**Table 4:** The effects of different dietary Met supplementation in low protein diets on the reproductive system of laying hens that are 38–50 weeks.

**Indexes**	**0.25%**	**0.31%**	**0.38%**	**0.47%**	**SE^c^**	***P*-value**	**Linear**	**Quadratic**
	**Met group**	**Met group**	**Met group**	**Met group**			***P-*value**	***P*-value**
Oviduct weight (g)	43.8^b^	49.7^a^^,^^b^	50.7^a^^,^^b^	55.3^a^	2.19	0.009	0.001	0.774
Oviduct length (cm)	67.9	73.9	71	71.6	2.4	0.383	0.444	0.278
Ovary weight (g)	33.1	34.3	35.6	42.3	2.5	0.061	0.014	0.28
Ovarian weight except the dominant follicles (g)	5.3	5.7	5.6	5.3	0.6	0.937	0.976	0.535
The number of rhubarb follicles	3.8	3.2	3.3	3.9	0.2	0.072	0.63	0.01
The number of small yellow follicles	1	1.2	1.2	1	0.1	0.227	1	0.04
The number of white follicles	0.9	0.9	0.9	1.4	0.29	0.455	0.214	0.352

a,b *Means within a column with no common superscripts differ (p < 0.05)*.

c *Pooled SEM*.

### Effects of Different Levels of Met Supplementation on the Gut Microbial Composition and Function of Laying Hens With Low Protein Diets

**Figure 1:**
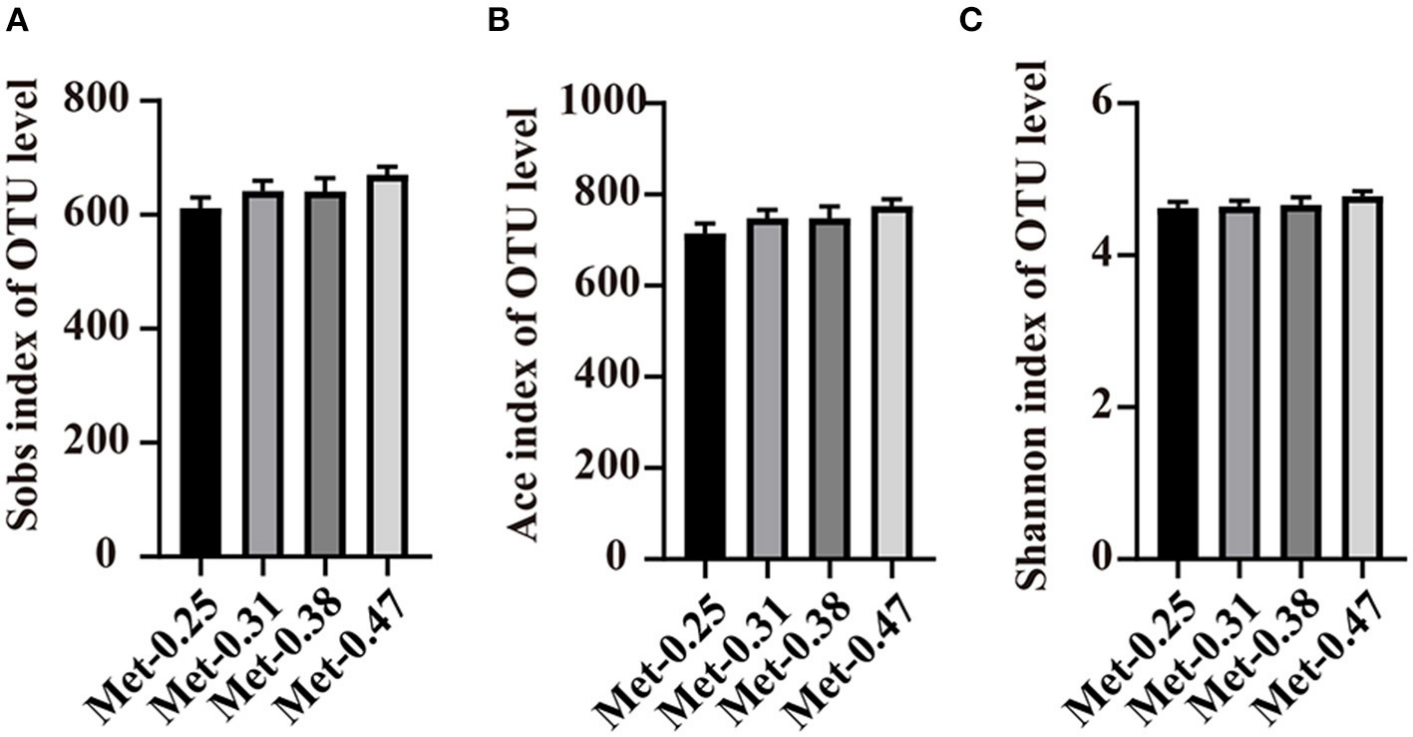
The effects of different dietary methionine (Met) supplementation in low protein diets on the alpha diversity of the cecal microbiota in laying hens. **(A)** Sobs index of the community diversity. **(B)** Ace index of the community richness. **(C)** Shannon index of the community diversity. Data were presented as means ± SEM (*n* = 9 per group). Significant differences were tested by a Student's *t*-test.

To investigate the effects of different Met levels in low protein diets on the gut microbial composition of laying hens, 16S rDNA sequencing was performed. In the end, 2,154,182 sequences were obtained. Through a clustering operation, the sequences were divided into many groups according to 97% similarity. Each group was an OTU, and a total of 1,075 OTUs were obtained, which could be divided into 19 phyla, 34 classes, and 174 genera. The results of the alpha diversity analysis showed that there was no significant difference between the Sobs index, ACE index, and Shannon index among the four treatment groups (Figures 1A–C).

**Figure 2:**
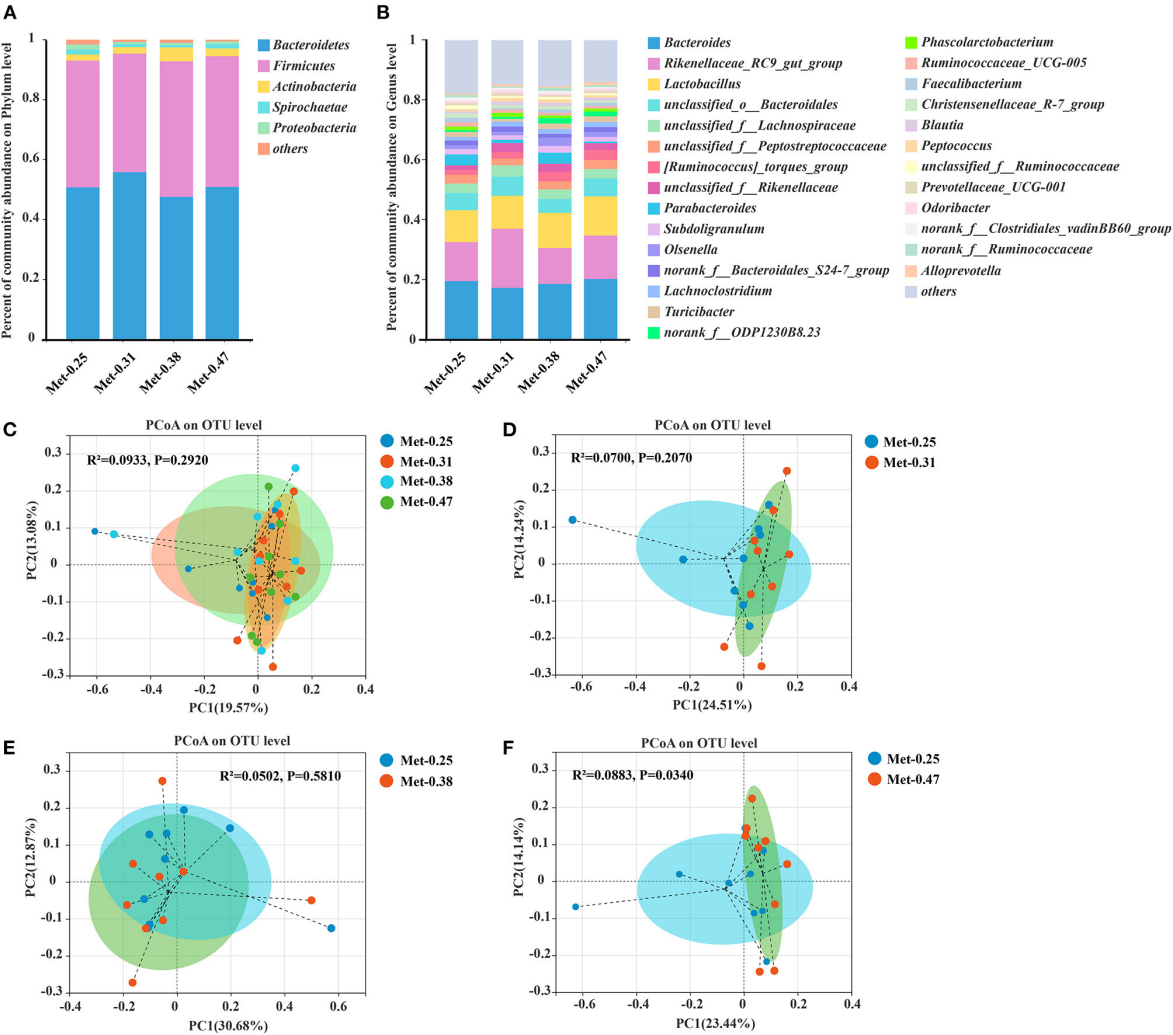
The effects of different dietary Met supplementation in low protein diets on the relative abundance in the cecal microbiota of laying hens and the principal coordinate analysis (PCoA) (Bray–Curtis distance) plot of the gut microbial community structure between the 0.25% Met group, 0.31% Met group, 0.38% Met group, and 0.47% Met group (*n* = 9 per group). **(A)** Relative abundance of gut microbiota at the phylum level (*n* = 9 per group). **(B)** Relative abundance of gut microbiota at the genus level (*n* = 9 per group). **(C)** The PCoA in the four treatments. **(D)** The PCoA in the 0.25 and 0.31% Met groups. **(E)** The PCoA in the 0.25 and 0.38% Met groups. **(F)** The PCoA in the 0.25 and 0.47% Met groups.

The relative abundance at the phylum and genus levels was studied. Notably, different levels of Met in diets changed the structure and composition of the intestinal microorganism of laying hens. From the perspective of the phylum level, *Bacteroidetes* and *Firmicutes* are the two kinds of bacteria that account for the largest proportion in the cecum of laying hens, exceeding 95% of the total cecum bacteria. In addition, the proportion of *Firmicute/Bacteroidetes* in the 0.25% Met group was similar to that in the 0.47% Met group, but both were lower than that in the 0.38% Met group and higher than that in the 0.31% Met group (Figure 2A). At the genus level, *Bacteroides, Rikenellaceae_RC9_gut_group, Lactobacillus*, and *Unclassified_O_Bacteroidales* accounted for nearly 50% of the total cecal bacteria. *Rikenellaceae_RC9_gut_group* was the highest in the 0.31% Met group, followed by the 0.47, 0.25, and 0.38% Met groups. The relative abundance of *Lactobacillus* in the cecum of laying hens also increased with the increase of dietary Met level (Supplementary Table 3). The proportions of *Bacteroides* in all treatment groups was similar, with these proportions in the 0.25, 0.31, 0.38, and 0.47% Met groups being 19.62, 17.37, 18.69, and 20.31%, respectively (Figure 2B). The results of the PCoA showed that the gut microbiota in the 0.25, 0.31, 0.38, and 0.47% Met groups were aggregated, respectively (Figure 2C). These results indicated that different dietary Met levels changed the gut microbiota structure of laying hens to some extent. Moreover, compared with the distribution of spots in the 0.25% Met group, the distribution of spots in the 0.31 and 0.47% Met groups was more dispersed, and the distribution pattern of spots in the 0.38% Met group was similar to that in the 0.25% Met group (Figures 2D–F).

**Figure 3:**
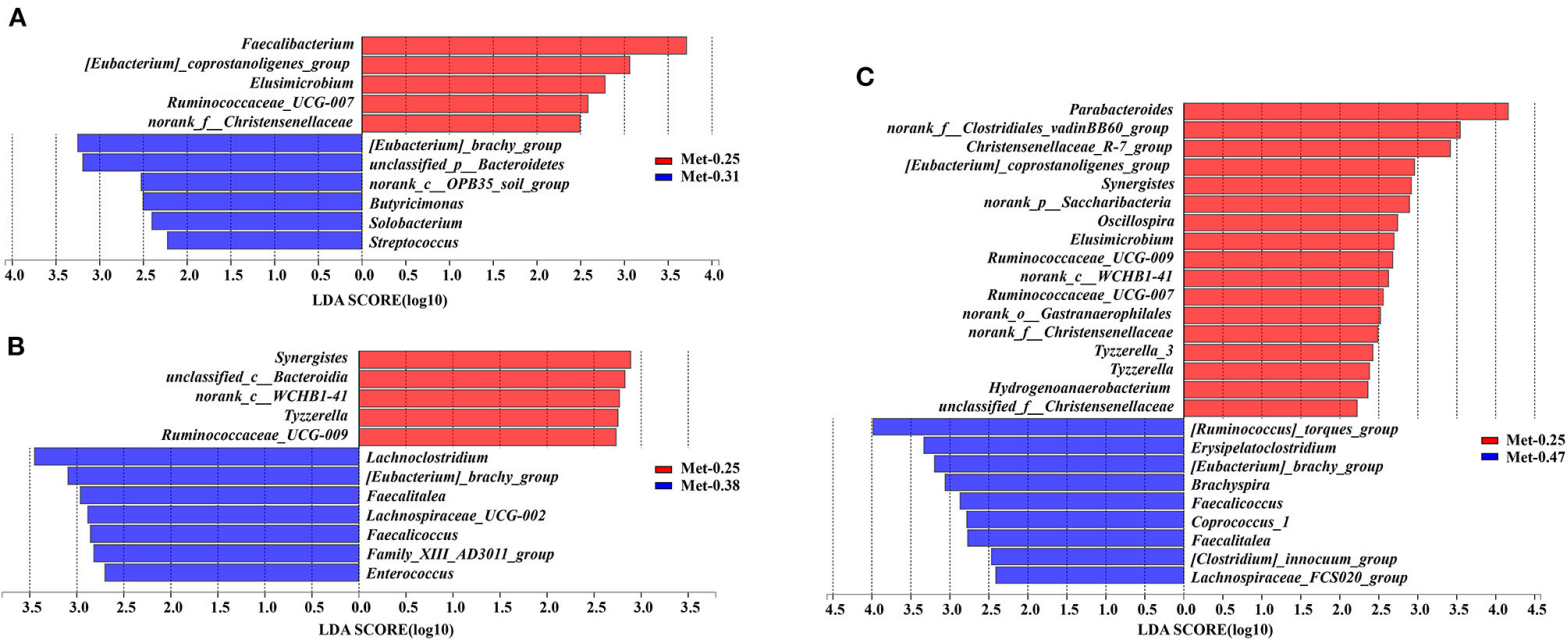
Differentially abundant genera in the gut microbiota of laying hens between the groups supplemented with different levels of Met. Histograms of the (linear discriminate analysis) LDA score (threshold ≥ 2) are plotted. An LDA effect size (LEfSe) was performed to determine the difference in abundance (*n* = 9 per group). **(A)** The LEfSe analysis of the gut microbiota in the 0.25 and 0.31% Met groups. **(B)** The LEfSe analysis of the gut microbiota in the 0.25 and 0.38% Met groups. **(C)** The LEfSe analysis of the gut microbiota in the 0.25 and 0.47% Met groups.

The LEfSe was used to figure out which bacterial taxa were statistically and biologically responsible for these differences. As shown in Figure 3, compared with the 0.25% Met group, *[Eubacterium]_brachy_group, unclassified_p_Bacteroidetes, norank_c_OPB35_soil_group, Butyricimonas, Solobacterium*, and *Streptococcus* were enriched in the 0.31% Met group (Figure 3A). *Lachnoclostridium, [Eubacterium]_brachy_group, Faecalitalea, Faecalicoccus, Lachnospiraceae_UCG-002, Family_XIII_AD3011_group*, and *Enterococcus* were enriched in the 0.38% Met group (Figure 3B). *[Ruminococcus]_torques_group, Erysipelatoclostridium, [Eubacterium]_brachy_group, Brachyspira, Faecalicoccus, Faecalitalea, Coprococcus_1, [Clostridium]_innocuum_group*, and *Lachnospiraceae_FCS020_group* were enriched in the 0.47% Met group (Figure 3C).

**Figure 4:**
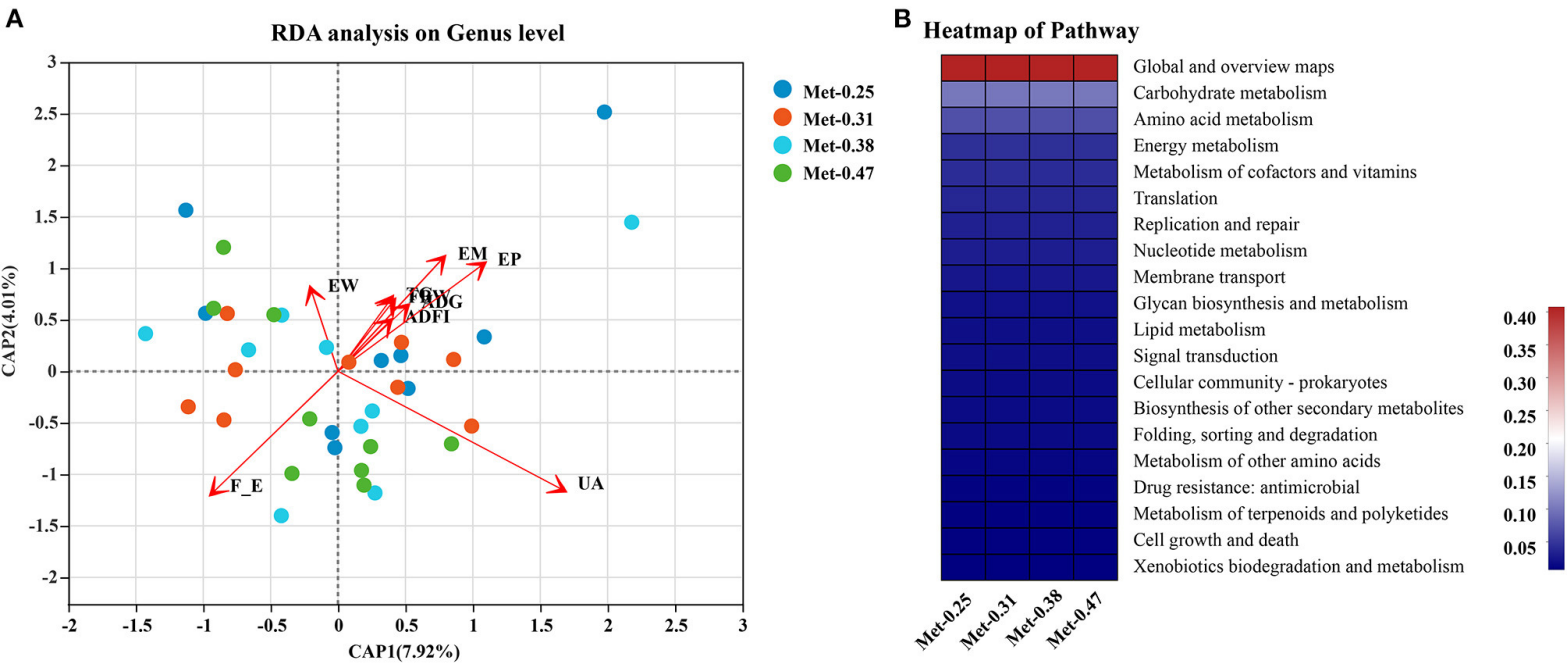
The effects of different dietary Met supplementation in low protein diets on the composition and function of gut microbiota of laying hens. **(A)** A redundancy analysis (RDA) of the distinctive genera and the phenotype of laying hens. **(B)** The 20 most significant Kyoto Encyclopedia for Genes and Genomes (KEGG) pathways upon different dietary Met supplementation in low protein diets.

As shown in Supplementary Figures 1A,B, the different levels of dietary Met have significant effects on the intestinal microorganisms of laying hens. For example, compared with the other three groups, the 0.25% Met group has more significant effects on *[Eubacterium]_coprostanoligenes_group, Ruminococcaceae_UCG-007, norank_f_Christensenellaceae, Elusimicrobium, Ruminococcaceae_UCG-009, norank_c_WCHB1-41*, and *norank_o_Gastranaerophilales*. In addition, *Ruminococcaceae_UCG-009* and *Norank_O_gastranaerophilales* were affected significantly, and the 0.31% Met group has more significant effects on [Eubacterium]_brachy_group. The 0.38% Met group has more significant effects on norank_p_Saccharibacteria, Faecalitalea, and *Family_XIII_AD3011_group*, while the 0.47% Met group has more significant effects on *Treponema_2, Coprococcus_1, Faecalicoccus, and [Clostridium]_innocuum_group* (Supplementary Figures 1A,B). To elucidate the underlying molecular mechanism of Met in a low protein diet on the gut microbial function of hens, a KEGG pathway analysis were performed (Figure 4B). Interestingly, the most enriched pathways were closely related to carbohydrate metabolism, amino acid metabolism, energy metabolism, metabolism of cofactors and vitamins, and lipid metabolism.

### Correlation Between the Differential Microbes Induced by the Supplementation of Met in Low Protein Diets and Key Parameters

**Figure 5:**
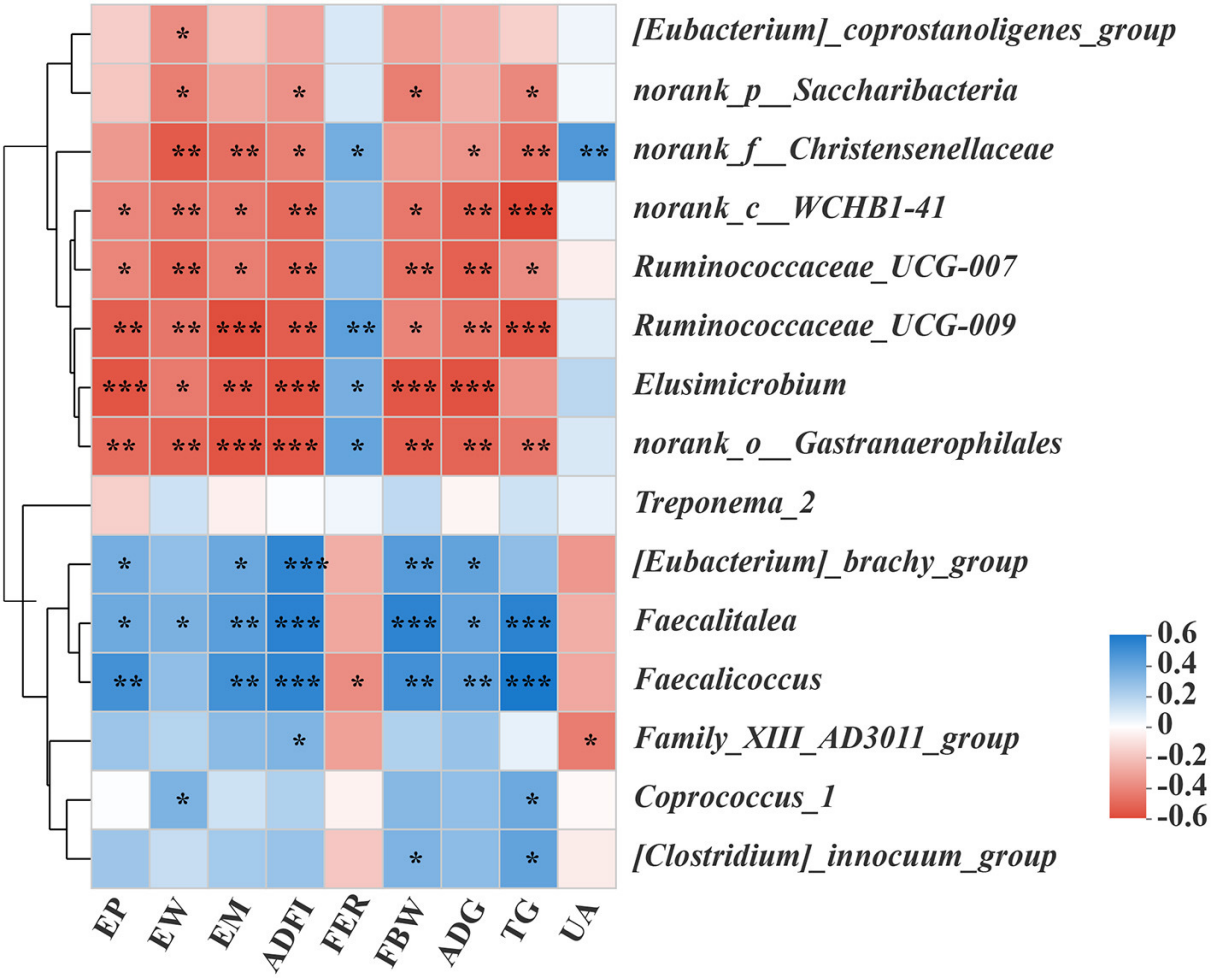
The effects of different dietary Met supplementation in low protein diets on the difference of the gut microbiota and its correlation with the phenotype of laying hens. The correlation between the various gut microbiota and the phenotype of laying hens. Asterisks indicate significant correlations (*p ≤ 0.05, **p ≤ 0.01, ***p ≤ 0.001). The blue represents a significantly positive correlation (*p* ≤ 0.05), the red represents a significantly negative correlation (*p* ≤ 0.05), and the white represents no significant correlation (*p* > 0.05).

In order to predict the correlation between the intestinal microbial communities and key parameters, an RDA and Spearman correlation matrix were performed. As shown in Figures 4A, 5, *Ruminococcaceae_UCG-007, Ruminococcaceae_UCG-00, norank_o_Gastranaerophilales*, and *norank_c_WCHB1-41* were positively correlated with ADFI, EW, FBW, TG, EM, EP, and ADG, among which *Ruminococcaceae_UCG-009* and *Norank_o_gastranaerophilales* were negatively correlated with FER (*p* < 0.05). On the contrary, *Faecalitalea* was negatively correlated with these traits (*p* < 0.05), while it was positively correlated with FER, but not significantly. *Elusimicrobium* was positively correlated with ADFI, EW, FBW, EM, EP, and ADG but negatively correlated with FER (*p* < 0.05). *Norank_f_Christensenellaceae* was significantly positively correlated with ADFI, EW, TG, EM, and ADG, but negatively correlated with FER and UA (*p* < 0.05). *Norank_P_Saccharibacteria* was significantly positively correlated with EW, ADFI, FBW, and TG (*p* < 0.05). *[Eubacterium]_coprostanoligenes_group* was significantly positively correlated with EW (*p* < 0.05). In contrast, *Faecalicoccus* was significantly negatively correlated with ADFI, FBW, TG, EM, EP, and ADG (*p* < 0.01), but positively correlated with FER (*p* < 0.05). *Family_xii_ad3011_group* was negatively correlated with ADFI, but positively correlated with UA (*p* < 0.05). *[Eubacterium]_Brachy_group* was negatively correlated with ADFI, FBW, EM, EP, and ADG (*p* < 0.05). *Coprococcus_1* was negatively correlated with EW and TG (*p* < 0.05). *[Clostridium]_innocuum_group* was significantly negatively correlated with FBW and TG (*p* < 0.05). There was a certain correlation between the production traits and other unmentioned intestinal microflora of laying hens, but their influence is not significant (Figure 5).

## Discussion

For a long time, Met has been used as a feed additive in livestock and poultry diets to maintain animal health and growth performance. According to previous studies, because of the lack of essential amino acids in low protein diets, production performance and egg quality are negatively affected, the intestinal morphology is damaged, and the immune response level is reduced (41–43). In this study, Peking Pink laying hens were selected as the experimental model to explore the effects of different levels of Met in the low protein diet on the performance, egg quality, serum biochemical indexes, and gut microbial composition of the laying hens. Our results showed that the supplementation of Met in the low protein diet could significantly improve various indices of laying hens.

When the dietary protein level is reduced to 13–14%, the performance of laying hens will be directly affected if the synthetic amino acids are not supplemented in time (44, 45). Keshavarz showed that the addition of synthetic amino acids to a low protein diet could effectively improve the performance of laying hens and reduce nitrogen emissions, which is also consistent with the results of others (46–48). From these, we can understand the significance of additional amino acids in the low protein diet, which is the same as the results in this experiment.

Studies have found that, if the Met intake of laying hens was increased, then EW would increase. Meanwhile, laying performance would be significantly improved, but with a decreased FER (19, 49). Similarly, we obtained the quadratic equation between Met and sulfur-containing amino acids and EP, EW, and FER. Within a certain range, the increase of Met level can increase EP and EM, while decreasing FER. These results indicated that the supplementation of Met in diets promotes the feeding and growth of laying hens and improves feed conversion ratio, which were also similar with Esteve (50). In our experiment, with the increase of Met, egg weight also increased at each stage. However, different Met levels had no significant effects on eggshell color, strength, Haugh unit, and other indices.

A number of indicators in the serum often reflect the health status of animals. When animals are in a healthy state, protein synthesis increases along with TP and ALB. In our experiment, TP and ALB in the 0.38 and 0.47% Met groups were higher than those in the other Met groups. The IgA in the 0.47% Met group was higher than that in the other three groups, which was similar to the research results of Liu et al. (51). Those indicated that the metabolism of laying hens in the 0.47% Met group was more vigorous, and the level of immune response was higher. As one of the end-products of protein metabolism in poultry, UA is usually used as an indicator to measure the requirement of amino acids. It reflects the level of protein metabolism in animals (52). Our results showed that the changes of UA were stable when the Met level was in the range of 0.31–0.47%, and UA was lower than that in the 0.25% Met group. Therefore, it could be inferred that the amino acids in laying hens in the three groups with higher Met levels were more fully utilized.

Dietary energy levels are the main nutritional factors affecting egg quality (53). Our results showed that there was a positive linear correlation between the dietary Met level and TG, which was similar to the difference in EW among all groups. We postulated that a higher TG in serum could synthesize more fat and fully meet the energy needs of laying hens, thus affecting EW. A certain amount of abdominal fat storage is beneficial to prevent insufficient energy intake from affecting laying performance (54), and, based on the results of the 0.25% Met group, we inferred that when the Met in the diet is insufficient, the liver metabolism of laying hens is disturbed and the fat transport is obstructed, leading to fatty liver disease in the end.

Normal and stable microflora is an important prerequisite to ensure the health of poultry. Intestinal microflora is involved in the metabolism and growth of the host, and also affects feed conversion and nutrient digestion and absorption by changing the intestinal tissue morphology of poultry, thus affecting their performance (55–57). Our results showed that, at the genus level, *Bacteroides, Rikenellaceae_RC9_gut_group, Lactobacillus*, and *unclassified_o_Bacteroidales* were the dominant genera accounting for nearly 50% of the total cecal bacteria of laying hens. In the meantime, the *Lactobacillus* proportion increased with the increase of Met. Yan used a metagenomic analysis technology to find that *Lactobacillus* could promote the absorption of nutrients and improve the feeding efficiency of poultry (58). Some scholars have found that *Lactobacillus* and *Bifidobacterium* can synthesize a variety of VB beneficial to animals by participating in their metabolism. It can also convert mineral elements into ions that are easily absorbed by animals to improve their utilization rate (59). In contrast to the change in the proportion of *Lactobacillus*, the proportion of *Faecalibacterium* decreases with the increase of Met in the diet. *Faecalitalea* and *Faecalicoccus* of *Clostridium* had significantly negative effects on ADFI, FBW, TG, EM, EP, ADG, and other indices, but had positive effects on FER. Other studies have shown that *Clostridia* has an adverse effect on animal performance (60), and our results are consistent with this finding. We can conclude that the supplementation of Met in diets is beneficial to laying hens. In conclusion, it can be inferred that Met in low protein diets may improve the intestinal morphology and production performance of laying hens by promoting beneficial bacteria proliferation and competitively inhibiting harmful bacteria proliferation or infection.

## Conclusion

The use of the low protein diet could alleviate the current situation of the raw material shortage of protein feed and reduce the nitrogen emission from the feces and urine of livestock and poultry to reduce environmental pollution. Adding Met to the low protein diet could have positive effects on the production performance, reproductive system, host metabolism, and gut microbial composition of laying hens. For example, the addition of Met increased the abundance of *Lactobacillus* and decreased the proportion of *Faecalibacterium* in the gut. Meanwhile, there were also significant correlations between the gut microbiota and traits of laying hens. Specifically, the proportion of *Faecalicoccus* was significantly positively correlated with FER, but negatively correlated with ADFI, FBW, ADG, and other traits. At present, there are few studies on the effects of low protein diets on the gut microflora of laying hens. We hoped that our study will fill in some gaps in this field.

## Data Availability Statement

The datasets presented in this study can be found in online repositories. The names of the repository/repositories and accession number(s) can be found at: https://www.ncbi.nlm.nih.gov/, PRJNA745253.

## Ethics Statement

The animal study was reviewed and approved by China Agricultural University Animal Care and Use Committee (AW32301202-2-1, Beijing, China).

## Author Contributions

MM, QM, JZ, and SG designed the study. MM, SG, and SH conducted the experiments, drafted the manuscript, polished the manuscript, and finished the submission. MM, ML, and SG guided the analysis of the experimental data. QM, LZ, and JZ helped with revisiting and reviewing the manuscript. All authors read and approved the final manuscript.

## Funding

This study was supported by the National Science Foundation of China (Grant No. 31772621), a Special Fund for China Agricultural Research System program (CARS-40-K08), National Key Research and Development Program of China (2017YFD0500500), and the Special Fund from Chinese Universities Scientific Fund (2018TC043).

## Conflict of Interest

The authors declare that the research was conducted in the absence of any commercial or financial relationships that could be construed as a potential conflict of interest.

## Publisher's Note

All claims expressed in this article are solely those of the authors and do not necessarily represent those of their affiliated organizations, or those of the publisher, the editors and the reviewers. Any product that may be evaluated in this article, or claim that may be made by its manufacturer, is not guaranteed or endorsed by the publisher.

## Supplementary Material

Supplementary Figure 1 The effects of different dietary Met supplementation in low protein diets on the difference of the gut microbiota of laying hens. **(A)** The LEfSe analysis of the gut microbiota in the four treatments. **(B)** The mean proportion of the gut microbiota in the four treatments.

Supplementary Table 1 Ingredients and nutrient content of the diets (% DM).

Supplementary Table 2 Regression equation between production performance and Met and sulfur-containing amino acid intake.

Supplementary Table 3 The proportion of gut microbiota within the four Met groups on genus level.

## Abbreviations

ADG: average daily gain
ADFI: average daily feed intake
AFY: yield of abdominal fat
ALB: albumin
BW: body weight
EP: egg production
EW: egg weight
EM: average daily egg mass
FBW: final body weight
FER: feed egg ratio
GSH: glutathione
IBW: initial body weight
IgM: immune globulin M
Met: methionine
SOD: superoxide dismutase
TG: triglyceride
TP: total protein
UA: uric acid.
