# Supplementary figures and images for: Effects of Different Methionine Levels in Low Protein Diets on Production Performance, Reproductive System, Metabolism, and Gut Microbiota in Laying Hens
Source: Front Nutr. 2021 Oct 6;8:739676. doi: 10.3389/fnut.2021.739676 (PMC8526799; doi:10.3389/fnut.2021.739676)

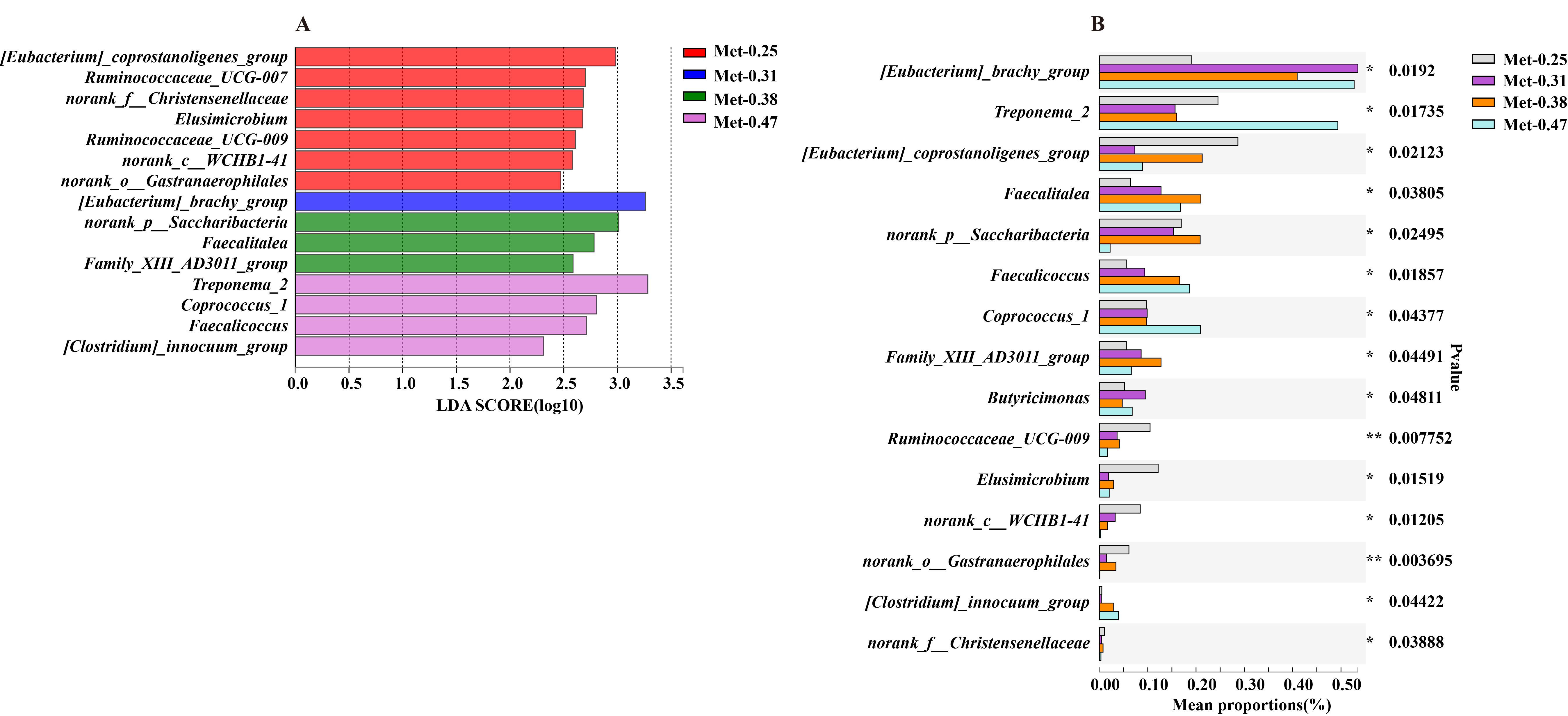

Supplement: Supplementary Figure 1 — The effects of different dietary Met supplementation in low protein diets on the difference of the gut microbiota of laying hens. (A) The LEfSe analysis of the gut microbiota in the four treatments. (B) The mean proportion of the gut microbiota in the four treatments. [file Image_1.TIF]
