# Supplementary material for: Effects of Different Methionine Levels in Low Protein Diets on Production Performance, Reproductive System, Metabolism, and Gut Microbiota in Laying Hens
Source: Front Nutr. 2021 Oct 6;8:739676. doi: 10.3389/fnut.2021.739676 (PMC8526799; doi:10.3389/fnut.2021.739676)
Supplement: Supplementary Table 1 — Ingredients and nutrient content of the diets (% DM). [file Table_1.docx]

**Table S1. Ingredients and nutrient content of the diets (% DM)**

| **Ingredients** | **0.25%**  **Met group** | **0.31%**  **Met group** | **0.38%**  **Met group** | **0.47%**  **Met group** |
| --- | --- | --- | --- | --- |
| Corn | 67.40 | 67.40 | 67.40 | 67.40 |
| Soybean meal | 12.40 | 12.40 | 12.40 | 12.40 |
| Peanut meal | 3.00 | 3.00 | 3.00 | 3.00 |
| Lysine hydrochloride | 0.24 | 0.24 | 0.24 | 0.24 |
| Met | 0.00 | 0.09 | 0.18 | 0.27 |
| Threonine | 0.10 | 0.10 | 0.10 | 0.10 |
| Valine | 0.04 | 0.04 | 0.04 | 0.04 |
| Isoleucine | 0.26 | 0.26 | 0.26 | 0.26 |
| Phenylalanine | 0.03 | 0.03 | 0.03 | 0.03 |
| Tryptophan | 0.03 | 0.03 | 0.03 | 0.03 |
| Soybean hull | 4.00 | 4.00 | 4.00 | 4.00 |
| Limestone | 8.30 | 8.30 | 8.30 | 8.30 |
| Dicalcium phosphate | 1.50 | 1.50 | 1.50 | 1.50 |
| NaCl (salt) | 0.30 | 0.30 | 0.30 | 0.30 |
| Vitamin premix ^1^ | 0.03 | 0.03 | 0.03 | 0.03 |
| Mineral premix^2^ | 0.30 | 0.30 | 0.30 | 0.30 |
| Choline chloride | 0.07 | 0.07 | 0.07 | 0.07 |
| Soybean oil | 1.00 | 1.00 | 1.00 | 1.00 |
| Zeolite powder | 1.00 | 0.91 | 0.82 | 0.73 |
| Total | 100.00 | 100.00 | 100.00 | 100.00 |
| **Nutrient^3^** | **0.25%**  **Met group** | **0.31%**  **Met group** | **0.38%**  **Met group** | **0.47%**  **Met group** |
| Crude protein | 13.23 | 13.31 | 13.40 | 13.49 |
| ME (Mcal/kg) | 2.70 | 2.70 | 2.70 | 2.70 |
| Met | 0.19(0.25) | 0.28(0.31) | 0.37(0.38) | 0.46(0.47) |
| Met + cysteine | 0.40(0.46) | 0.49(0.52) | 0.58(0.59) | 0.66(0.68) |
| Lysine | 0.76 | 0.76 | 0.76 | 0.76 |
| Tryptophan | 0.16 | 0.16 | 0.16 | 0.16 |
| Threonine | 0.56 | 0.56 | 0.56 | 0.56 |
| Arginine | 0.84 | 0.84 | 0.84 | 0.84 |
| Histidine | 0.34 | 0.34 | 0.34 | 0.34 |
| Isoleucine | 0.72 | 0.72 | 0.72 | 0.72 |
| Leucine | 1.17 | 1.17 | 1.17 | 1.17 |
| Phenylalanine | 0.63 | 0.63 | 0.63 | 0.63 |
| Phenylalanine + tyrosine | 1.08 | 1.08 | 1.08 | 1.08 |
| Valine | 0.60 | 0.60 | 0.60 | 0.60 |
| Glycine + Serine | 1.06 | 1.06 | 1.06 | 1.06 |
| Bird digestible lysine | 0.64 | 0.64 | 0.64 | 0.64 |
| Total P | 0.54 | 0.54 | 0.54 | 0.54 |
| Non-phytate phosphorus | 0.36 | 0.36 | 0.36 | 0.36 |
| Na | 0.15 | 0.15 | 0.15 | 0.15 |
| Cl | 0.26 | 0.26 | 0.26 | 0.26 |
| Ca | 3.54 | 3.54 | 3.54 | 3.54 |

^1^ Vitamin premix supplied (per kg of diet): Vitamin A, 96 000 IU; Vitamin D3, 3 600 IU; Vitamin E, 75mg; Vitamin K3, 4.8 mg; Vitamin B1, 4.8 mg; Vitamin B2, 9 mg; Folic acid, 0.9 mg; Calcium pantothenate, 15 mg; Niacin 45 mg; Vitamin B6, 4.4 mg; Vitamin B12, 24 μg; Biotin: 0.15 mg.

^2^ Mineral premix provided (per kg of diet): Cu, 6.8 mg; Fe, 66 mg; Zn, 83 mg; Mn, 80 mg; I, 1 mg; Se, 0.3 mg.

^3^ The nutrient levels were calculated values, measured values are shown in brackets.
