# Supplementary material for: Effects of Different Methionine Levels in Low Protein Diets on Production Performance, Reproductive System, Metabolism, and Gut Microbiota in Laying Hens
Source: Front Nutr. 2021 Oct 6;8:739676. doi: 10.3389/fnut.2021.739676 (PMC8526799; doi:10.3389/fnut.2021.739676)
Supplement: Supplementary Table 2 — Regression equation between production performance and Met and sulfur-containing amino acid intake. [file Table_2.docx]

**Table S2. Regression equation between production performance and methionine and sulfur-containing amino acid intake.**

| **Indexes** | **Equation** | ***X* (g/d)** | **Met level(%)** | ***Y*** | ***R²*** |
| --- | --- | --- | --- | --- | --- |
| EP/% | *Y* = -468.81x^2^ + 424.37x - 7.2022 | 0.45 | 0.38 | *Y*_max_=88.83 | 0.994 |
| EM g/d | *Y* = -328.41x^2^ + 303.05x - 15.589 | 0.46 | 0.41 | *Y*_max_*=*54.32 | 0.980 |
| FER | *Y* = 10.106x^2^ - 9.3686x + 4.2153 | 0.46 | 0.41 | *Y*_min_*=*2.04 | 0.992 |
| **Indexes** | **Equation** | ***X* (g/d)** | **Sulfur-containing amino acid level(%)** | ***Y*** | ***R²*** |
| EP/% | *Y* = -348.56x^2^ + 479.83x - 76.858 | 0.69 | 0.62 | *Y*_max_=88.28 | 0.999 |
| EM g/d | *Y* = -243.09x^2^ + 340.25x - 65.084 | 0.70 | 0.63 | *Y*_max_*=*53.98 | 0.992 |
| FER | *Y* = 7.4094x^2^ - 10.422x + 5.7198 | 0.70 | 0.63 | *Y*_min_*=*2.05 | 0.998 |
