# Supplementary material for: Effects of Different Methionine Levels in Low Protein Diets on Production Performance, Reproductive System, Metabolism, and Gut Microbiota in Laying Hens
Source: Front Nutr. 2021 Oct 6;8:739676. doi: 10.3389/fnut.2021.739676 (PMC8526799; doi:10.3389/fnut.2021.739676)
Supplement: Supplementary Table 3 — The proportion of gut microbiota within the four Met groups on genus level. [file Table_3.docx]

**Table S3. The proportion of gut microbiota within the four methionine groups on genus level**

| **OTU ID** | **0.25%**  **Met group** | **0.31%**  **Met group** | **0.38%**  **Met group** | **0.47%**  **Met group** |
| --- | --- | --- | --- | --- |
| *Bacteroides* | 0.19616 | 0.17366 | 0.18691 | 0.20306 |
| *Rikenellaceae_RC9_gut_group* | 0.13008 | 0.19720 | 0.11976 | 0.14545 |
| *Lactobacillus* | 0.10666 | 0.11009 | 0.11727 | 0.13068 |
| *unclassified_o_Bacteroidales* | 0.05729 | 0.06336 | 0.04639 | 0.05997 |
| *unclassified_f_Lachnospiraceae* | 0.03152 | 0.03867 | 0.03178 | 0.03114 |
| *unclassified_f_Peptostreptococcaceae* | 0.02919 | 0.02224 | 0.02664 | 0.03031 |
| *[Ruminococcus]_torques_group* | 0.01593 | 0.02252 | 0.03208 | 0.03404 |
| *unclassified_f_Rikenellaceae* | 0.01540 | 0.02912 | 0.02732 | 0.02155 |
| *Parabacteroides* | 0.03654 | 0.01112 | 0.03680 | 0.00542 |
| *Subdoligranulum* | 0.01785 | 0.01476 | 0.02226 | 0.01580 |
| *Olsenella* | 0.01303 | 0.01146 | 0.02736 | 0.01533 |
| *norank_f_Bacteroidales_S24-7_group* | 0.01571 | 0.01790 | 0.01287 | 0.01727 |
| *Lachnoclostridium* | 0.01226 | 0.01653 | 0.01670 | 0.01722 |
| *Turicibacter* | 0.01643 | 0.00856 | 0.01784 | 0.01873 |
| *norank_f_ODP1230B8.23* | 0.00609 | 0.00686 | 0.01850 | 0.01587 |
| *Phascolarctobacterium* | 0.01177 | 0.01388 | 0.00861 | 0.01019 |
| *Ruminococcaceae_UCG-005* | 0.01441 | 0.01038 | 0.00911 | 0.00849 |
| *Faecalibacterium* | 0.01540 | 0.00546 | 0.01084 | 0.01051 |
